# The degradation of gelatin/alginate/fibrin hydrogels is cell type dependent and can be modulated by targeting fibrinolysis

**DOI:** 10.3389/fbioe.2022.920929

**Published:** 2022-07-22

**Authors:** Elea Boucard, Luciano Vidal, Flora Coulon, Carlos Mota, Jean-Yves Hascoët, Franck Halary

**Affiliations:** ^1^ Nantes Université, INSERM, Center for Research in Transplantation and Translational Immunology, UMR 1064, Nantes, France; ^2^ Rapid Manufacturing Platform, Institut de Recherche en Génie Civil et Mécanique (GeM), UMR 7 CNRS 6183 Ecole Centrale de Nantes, Nantes, France; ^3^ Department of Complex Tissue Regeneration, MERLN Institute for Technology-inspired Regenerative Medicine, Maastricht University, Maastricht, Netherlands

**Keywords:** gelatin, sodium alginate, fibrinolysis, fibroblasts, aprotinin, matrix metalloproteinases, connective tissue, tissue engineering

## Abstract

In tissue engineering, cell origin is important to ensure outcome quality. However, the impact of the cell type chosen for seeding in a biocompatible matrix has been less investigated. Here, we investigated the capacity of primary and immortalized fibroblasts of distinct origins to degrade a gelatin/alginate/fibrin (GAF)-based biomaterial. We further established that fibrin was targeted by degradative fibroblasts through the secretion of fibrinolytic matrix-metalloproteinases (MMPs) and urokinase, two types of serine protease. Finally, we demonstrated that besides aprotinin, specific targeting of fibrinolytic MMPs and urokinase led to cell-laden GAF stability for at least forty-eight hours. These results support the use of specific strategies to tune fibrin-based biomaterials degradation over time. It emphasizes the need to choose the right cell type and further bring targeted solutions to avoid the degradation of fibrin-containing hydrogels or bioinks.

## Introduction

The oral cavity is lined with mucosal tissues which protect from physical, chemical, thermal and microbial threats (Presland and Dale, 2000). Like skin, oral mucosae are defined as composite tissues with a typical two-layer architecture: a connective tissue (CT) or *lamina propria* which consists in an extracellular matrix (ECM) containing stromal cells, e.g., fibroblasts or endothelial cells, supporting a squamous pluristratified epithelium mainly composed of specialized keratinocytes (Presland and Dale, 2000; Stecco et al., 2015). Both layers are separated by a basement membrane (Kinikoglu et al., 2015). In general, the CT ECM is a complex network surrounding cells, mostly composed of proteins, e.g., collagens and elastin, glycoproteins proteoglycans and glycosaminoglycans (Frantz et al., 2010; Skardal et al., 2015; Naba et al., 2016). It undergoes a constant cell-mediated remodeling most notably via matrix metalloproteinases or MMPs (Frantz et al., 2010; Bonnans et al., 2014).

Tissue engineering (TE) has allowed recapitulating part of the complexity of human skin and mucosal tissues through the development of 3D models *in vitro* as well as, more recently, *in vivo* (Albouy et al., 2022). The current advantages of *in vitro* 3D models are: they mimic the structure and functions of their native counterparts, they support the use of genetically-modified cell lines, they can be fully humanized, and they comply with the reduction in animal testing. To date, manual-, e.g., with cell culture inserts (Reijnders et al., 2015; Buskermolen et al., 2016), and automated engineering, e.g., bioplotting (Pourchet et al., 2017), of humanized CT equivalents (CTE) have already been described. Biomaterials frequently used for CTE biofabrication are natural-based (e.g., collagen, gelatin, fibrinogen, hyaluronic acid, and alginate) or synthetic polymers (e.g., PEG, PLGA) (Urciuolo et al., 2019) of various stiffness (Skardal et al., 2015; Pepelanova et al., 2018; Vidal et al., 2020) as single component or blended hydrogels (Skardal et al., 2012; Skardal et al., 2015). Furthermore, to select appropriate hydrogels needs to consider other parameters such as the cytocompatibility and remodeling, mainly driven by a fine balance in ECM degradation versus neosynthesis (Caliari and Burdick, 2016). A 3D bioprinted dermis equivalent, made of gelatin (GL), sodium alginate (SA) and fibrin (FB), was recently described by Pourchet et al. (2017). A thermosensitive mixture of degraded ECM, GL, was used to maintain shape fidelity upon printing. GL was mixed with SA, which improves the scaffold stability after cross-linking, and fibrinogen (FBG), a secreted blood glycoprotein responsible for blood clots formation, leading to FB fibers formation after cleavage by thrombin. Both GL and FB harbor RGD motifs to promote cell attachment in the 3D network formed by the three distinct compounds after cross-linking. Likewise, similar blended hydrogels were shown to support the biofabrication of 3D models of metabolic syndrome (Xu et al., 2010), cervical and brain cancers (Zhao et al., 2014; Dai et al., 2016), cartilage implants (Henrionnet et al., 2020) and oral mucosa regeneration (Yi et al., 2022). Fibroblasts are the most abundant cells in CT under physiological conditions although their proportion may vary according to the tissue location. They are key players in the CT homeostasis, displaying extensive heterogeneity, plasticity and exhibiting various functions like remodeling ECM, providing signals essential to epithelial cell survival, proliferation and differentiation (Merne and Syrjänen, 2003; Okazaki et al., 2003; Lebleu and Neilson, 2020; Tsukui et al., 2020; Buechler et al., 2021; Reynolds et al., 2021) or promoting angiogenesis, immune control and tissue regeneration (Lebleu and Neilson, 2020). Human primary and hTERT-immortalized fibroblasts have already been used successfully in CTE reconstruction based on GL/SA/FB hydrogels, termed GAF in this study (Pourchet et al., 2017; Henrionnet et al., 2020). However, their ability to remodel GAF blends has not been clearly addressed to date.

Our study aimed at assessing the capacity of primary and immortalized human fibroblasts of distinct origins to degrade GAF hydrogels. We confirmed the efficacy of the well-known aprotinin, a serine protease inhibitor, in controlling fibrinolysis (Lorentz et al., 2010; Lesman et al., 2011; Thomson et al., 2014). However, we showed that human, plasminogen-depleted fibrinogen, mixed with GL and SA, leads to less GAF degradation compared to human or even bovine fibrinogens. Both can be used alone or simultaneously to tune fibrinolysis thus achieving stable, bioengineered CTEs.

## Materials and methods

### Reagents

Collagenase IV from *Clostridium histolyticum*, porcine gelatin (GL), fibrinogen (FBG) from bovine or human origin, dehydrated calcium chloride (C7902), thrombin from bovine plasma, Marimastat ((2S, 3R)-N-[(1S)-2,2-dimethyl-1-(methylcarbamoyl)propyl]-N′,2-dihydroxy-3-(2-methylpropyl)butanediamide) were purchased from Sigma-Aldrich (Saint-Louis, MI). Sodium alginate (SA) was obtained from Alfa Aesar (Haverhill, MA). Human plasminogen-depleted fibrinogen was utilized for hydrogel preparation when stated (Merck, Darmstadt, Germany). Aprotinin was purchased from R&D Systems (Minneapolis, MI). All reagents for cell culture were purchased from Gibco (ThermoFisher Scientific, Waltham, MA).

### Isolation of human primary fibroblasts

Primary human gingival (HGF) or foreskin (FSF) fibroblasts were isolated from adult tissues. Samples were obtained from healthy donors undergoing surgery under informed consent. Surgical discards were stored in Hanks’ Balanced Salt Solution supplemented with 200 U/ml of penicillin, 200 μg/ml of streptomycin and 2.5 μg/ml amphotericin B, later referred to as antibiotics (ABX), and processed within 24 h post-sampling. Biopsies were washed three times in ABX-containing dPBS 1X and incubated overnight at 4°C in trypsin 0.05% to detach epithelium from the CT enzymatically. Subsequently, epithelial sheets were gently separated from CT with fine forceps. Epithelium-free CTs were digested in a mixture of DMEM and 2 mg/ml collagenase IV at 37°C for 1 h and vortexed vigorously every 15 min. HGF primary cultures were also kindly gifted by Dr Philippe Lesclous (Université de Nantes, CHU Hôtel Dieu, INSERM U1229, RMeS). FSF were isolated from fresh biopsies. Briefly, after mechanical removal of the hypodermis and deep dermis, samples were cut into pieces of 4 mm^2^ and incubated 2 h at 37°C in dispase 1X (Invitrogen, Cergy Pontoise France). Epidermis and dermis were dissociated with forceps. Dermal pieces were incubated in RPMI supplemented with 10% FBS in 24-well plates under standard cell culture conditions with ABX. Culture medium was replaced every 2–3 days until FSF migrated out and started to proliferate, usually between day 10 and 14. At confluency, FSF were harvested and cultivated in culture flasks or frozen until use.

### Cell culture

HGF, FSF and embryonic lung fibroblasts MRC-5 (RD-Biotech, Besançon, France) were expanded in DMEM supplemented with, 10% FBS, 2 mM L-glutamine and ABX. Cells were cultured in a humidified incubator at 37°C in 5% CO_2_. Medium was changed every 2–3 days and cell passages were carried out using a TrypLE Express solution (Gibco). Human TERT-immortalized gingival fibroblasts (hTERT-HGF, CRL-4061, ATCC, London, United Kingdom) were cultivated according to the manufacturer’s instructions.

### In-gel 3D cell culture

GAF was formulated as previously described by Pourchet et al. (2017). Briefly, GL, SA and FBG were dissolved in 0.9% NaCl at 37°C. Alternatively, GL/SA- or FBG-based hydrogels were obtained by mixing the respective compounds with MRC-5 cells. In all hydrogel formulations, cells were seeded at 1 × 10^6^ cells/mL as described elsewhere (Almela et al., 2016; Pourchet et al., 2017). Equivalent cell quantities were seeded onto cell culture treated 6-well plates as 2D culture controls. All hydrogel formulations were poured into 12-well, 3 µm pore-sized PET membrane culture inserts (Falcon, Corning, NY). Crosslinking of GAF and GL/SA gels was performed by incubating the conditions for 30 min at 25°C in 100 mM CaCl_2_ (GL/SA gels) and an additional 20 U/mL of thrombin (GAF). FBG gels were produced by mixing thrombin solution to the FBG and immediately poured into the inserts. All gels were washed in 0.9% NaCl and subsequently placed under standard cell culture conditions.

### Gel degradation assessment

Gel degradation was assessed by measuring white light diffusion through manually-deposited GAF cast in culture inserts (3 mm thick). The experimental setup was made of a LED light source (4000 K) and a digital microscope (Dino-Lite, Taiwan) as an image collector. Mean grey values of five separate regions of interest (ROIs) of each gel were normalized over DMEM alone (set as 100% degradation) or GAF without cells (set as no degradation). The results are displayed as the percentage of degradation. Proteasomal inhibition was performed on MRC-5-laden gels using aprotinin (20 or 40 μg/ml) or Marimastat (100 µM). MRC-5 cells were resuspended in NaCl 0.9% containing tested drugs and seeded in GAF at 1 × 10^6^ cells/mL. Culture medium was supplemented with tested drugs as well for the duration of the assay. Degradation was assessed after 48 h of culture at 37°C, 5% CO_2_ unless otherwise stated.

### Gene expression analysis

2D-cultured cells were detached using TrypLE Express and pelleted by centrifugation. Total RNA extraction was performed on cell pellets with TRIzol Reagent (Invitrogen) according to the manufacturer’s guidelines. RNA precipitation was performed overnight at −20°C, with absolute ethanol. Quality of total RNAs was determined by absorbance ratios at 260 nm/280 nm using a NanoDrop 2000 spectrophotometer (ThermoFisher). Single-stranded cDNA synthesis was performed from 1 µg of RNA with M-MLV reverse transcriptase (ThermoFisher Scientific). Reverse transcription (RT) was performed using the following thermocycling conditions: 72°C for 10 min, 37°C for 1 h and −20°C for storage until use. Quantitative PCR amplifications were performed on a Viia7 thermal cycler (ThermoFisher Scientific) in 10 µl reaction mix containing 5 µl of Taqman Buffer 2X, 0.5 µl of probes, 2.5 µl of sterile water and 2 µl sample diluted 1:5 in sterile water. Expression level of genes coding for Epidermal growth factor receptor (*EGFR*), Vimentin (*VIM*), S100 calcium binding protein A4 (*S100A4*), Fibroblast Activation Protein Alpha (*FAP*), Short stature homeobox 2 (*SHOX-2*), Thy-1 cell surface antigen (*THY-1*), amine oxidase, copper containing 3 (*AOC-3*)*,* ɑ-smooth muscle actin (*ACTA2*), platelet-derived growth factor receptor A (*PDGFRA*) and type I collagen (COL1A1) (Thermo Fisher, Waltham, Massachusetts) was investigated. Probe references, amplicon sizes and target genes are listed in Supplementary Table S1. The housekeeping gene *GAPDH* (Glyceraldehyde 3-phosphate dehydrogenase) was used as a reference gene. All runs were performed in duplicates.

### Secreted protease analysis

After 48 h, supernatants from MRC-5 and hTERT-HGF 2D and 3D cultures were harvested, pooled and filtered through a 100 µm filter (3D condition). Supernatants were centrifuged at 2500 rpm for 5 min to remove insoluble material and stored at −80°C until use. Gels without cells or containing hTERT-HGF were incubated with supernatants from 3D MRC-5 cultures (condition medium, MRC-5 CM) when required. Soluble protease expression profile of MRC-5 and hTERT-HGF 2D and 3D conditions were performed using a Human Protease Array kit according to the manufacturer’s guidelines (R&D Systems, Minneapolis, MI). Images were acquired on a LAS-4000 Fujifilm imager and analyzed after subtraction of background using “Protein Array Analyzer” plugin in (Carpentier and Henault, 2010) ImageJ software. The mean pixel density of analyte spots was normalized on reference spots (100%) and negative controls (0%) mean pixel density for each membrane. Results are expressed as Mean Pixel Density (MPD) ± SD. The threshold for significance was inferred from background values and set up at 15%.

### Confocal imaging of GL/SA and fibrinogen hydrogels

GL/SA and FBG gels were stained using calcein AM (04511, Supelco, Bellefonte, Pennsylvania, United States) according to manufacturer’s instructions. Each sample was placed on a coverslip and subsequently imaged using a Nikon A1-Rsi confocal microscope equipped with Nikon Plan Fluor 10×0.30 NA objective. Image stacks were acquired and treated using NIS-Elements AR software. All acquisitions were performed using the same parameters. Poisson shot noise was removed using the deep learning *Denoise* algorithm*.* Images are represented as a volume view using Depth Coded Alpha Blending (color gradient perpendicular to the Z-stacks).

### Statistical analysis

Statistical analyses were performed by GraphPad Prism 8. *p*-values below 0.05 were considered as statistically significant. Results are expressed as mean ± SD. For degradation assays and proteome profile analysis, two-way ANOVA tests were used. Comparison of cell types using RT-qPCR was conducted by the Kruskal–Wallis test.

## Results

### Fibroblasts display different abilities to degrade the gelatin/alginate/fibrin hydrogel

**FIGURE 1:**
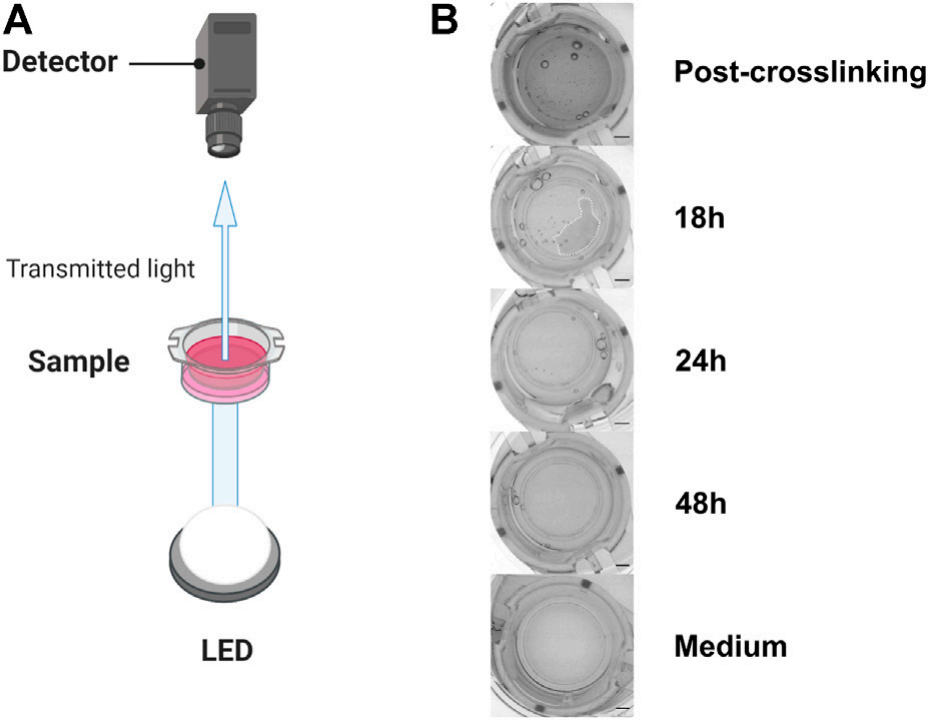
Experimental setup to evaluate GAF degradation **(A)**. **(B)** From top to bottom, typical images of culture inserts containing GAF with MRC-5 cells right after crosslinking (Post-crosslinking), 18 (partial GAF degradation, degradation foci are visible), 24 and 48 h post-crosslinking showing the rapid GAF degradation kinetics and without hydrogel (Medium). Scale bar, 2 mm. Created with BioRender.com.

**FIGURE 2:**
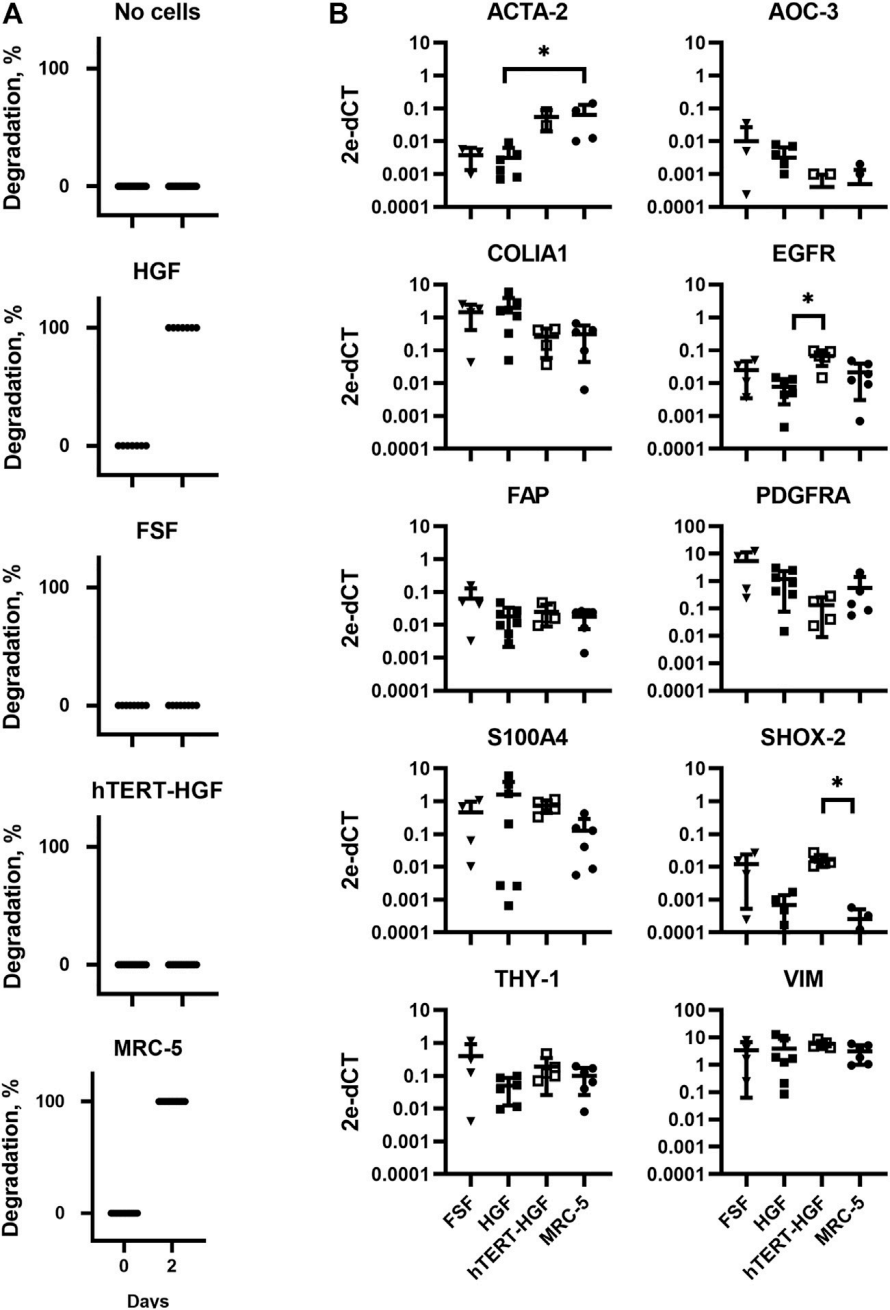
Assessment of GAF degradation by HGF, FSF, immortalized HGF (hTERT-HGF) and MRC-5 cells two days post-crosslinking. **(A)** Degradation at day zero, i.e., right after GAF crosslinking, is the non-degradation control for each cell type. Individual values are plotted (*n* ≤ 16). **(B)** Gene expression analysis in FSF, HGF, hTERT-HGF and MRC-5 cells for ACTA-2, AOC-3, FAP, EGFR, COLIA1, THY-1, PDGFRA, SHOX-2 and S100A4 genes in standard 2D in vitro cultures by RT-qPCR. HGF and FSF were isolated from three independent donors; mean values are displayed for all samples. Values were normalized on RFPL0. Statistically significant results were marked by one or several asterisks according to the level of significance: *p < 0.05; Kruskal-Wallis tests.

In order to fabricate a stable model of CTE to support further viral infection studies we first sought to characterize appropriate primary fibroblasts or immortalized fibroblastic cell lines of various origins, namely HGF and hTERT-HGF (oral mucosa), FSF (foreskin) and MRC-5 cells (embryonic lung). To that purpose, each cell type was seeded in a GAF hydrogel and immersed in culture medium for 48 h. Gel stability was then monitored by quantifying transmitted white light through hydrogels by image analysis thanks to a home-made image collection system (Figure 1). We observed that GAF alone, or containing hTERT-HGF and FSF showed no degradation for at least 48 h in culture. Conversely, MRC-5 cells and HGF demonstrated a rapid degradation compared to the cell-free control (Figure 2A). We wondered whether these differences in GAF degradation abilities between cell types could be linked to different gene expression known to segregate between resting and activated fibroblasts or even myofibroblastic cells like cancer-associated fibroblasts (CAFs), all being known to have quite distinct ECM-degradation abilities. The expression of *EGFR, VIM, FAP, THY-1, S100A4, SHOX-2, AOC-3, ACTA2*, *PDGFRA,* and *COL1A1* in HGF, FSF, hTERT-HGF and MCR-5 was assessed (Figure 2B; Supplementary Figure S1). The four cell types express moderate to high levels of *COL1A1, VIM, THY-1* and *PDGFRA.* Conversely, they all express low amounts of *AOC-3* and *FAP* RNAs, typical of myofibroblasts or CAFs. Both observations confirmed a well-established resting or weakly activated fibroblast signature. Significant differences were noted between HGF and MRC-5 for *ACTA-2* or in *EGFR* differential expression between primary and hTERT-immortalized HGF but without obvious correlation with their GAF-degradation status. Interestingly, *SHOX-2* was shown to be expressed at higher levels in cells unable to degrade hydrogels, the FSF and hTERT-HGF (Figure 2B; Supplementary Figure S1). Altogether, these results demonstrated that the four tested cell types harbor a resting fibroblast signature but no clear RNA expression profile compatible with an ECM degradation program.

### MRC-5 cells degrade fibrin-based hydrogels

**FIGURE 3:**
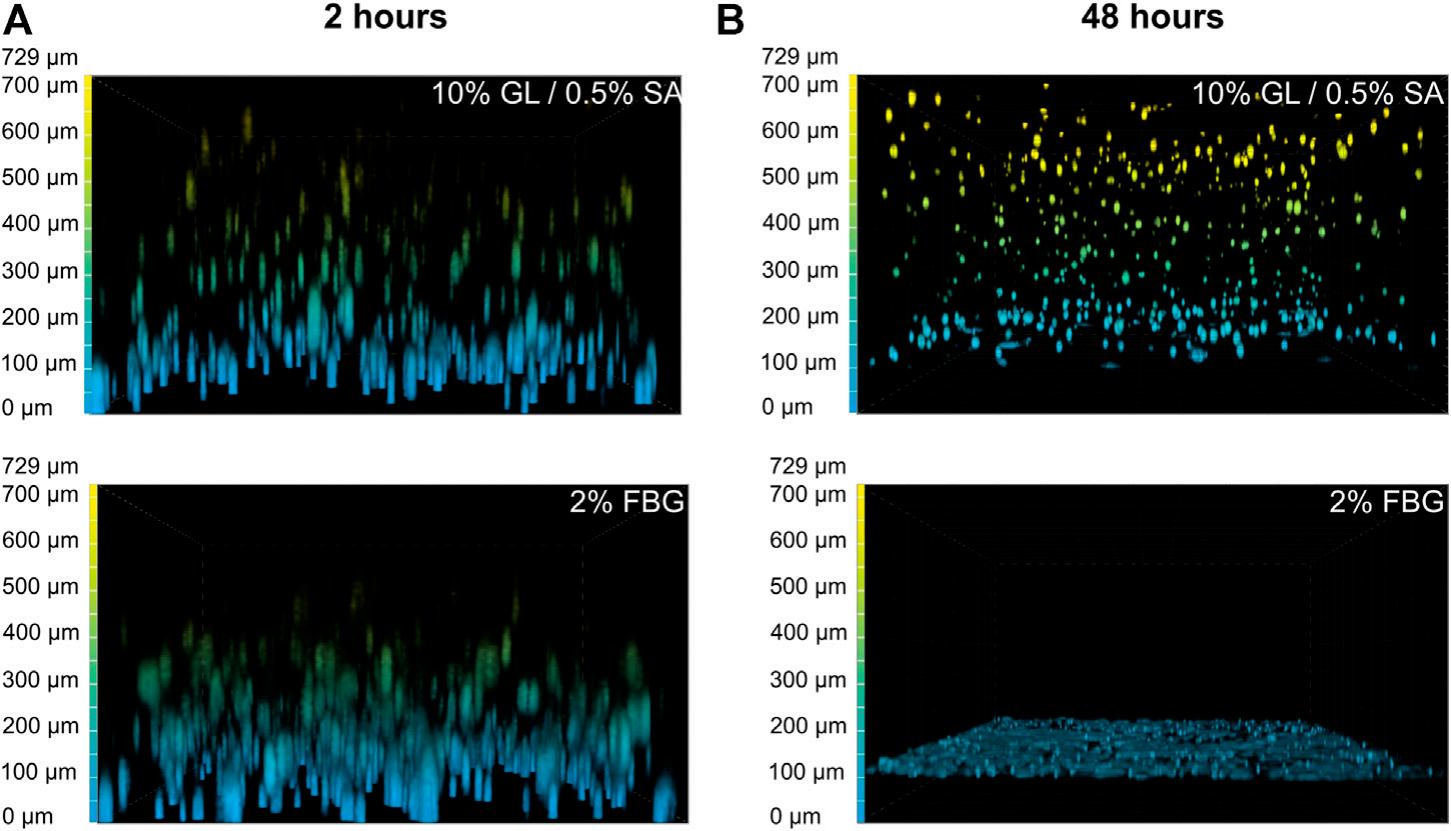
GAF degradation by MRC-5 cells results from fibrin network disruption. MRC-5 cell-seeded GL/SA or FBG crosslinked matrices in culture inserts were analyzed by confocal microscopy (×10 objective) after 2 h **(A)** or 24 h **(B)**. Cells were labeled with calcein AM prior to image acquisition. Images are represented as false-colored by Depth Coded Alpha Blending on NIS-Elements AR, i.e., blue-colored cells are close or adherent to the membrane of culture inserts while yellow-colored cells are the most distant.

To figure out why MCR-5 cells were actively degrading GAF hydrogels, we first investigated which component of the GAF was targeted during degradation. GL provides stability during the formulation of the hydrogel but has been shown to progressively leak out of the hydrogel at least for low-molecular weight GL fractions whereas SA and FBG provide a dual structural network. As some fibroblasts are known to exhibit gelatinase or fibrinolytic activities, MRC-5 were seeded in either GL/SA (10:0.5%) or 2% FBG 3D scaffolds. Confocal 3D reconstructions showed that both GL/SA and FBG hydrogels contained fluorescent-labeled cells homogeneously spreading throughout the gel volume, i.e., from the membrane up to approximately 700 µm in-gel towards the hydrogel surface in the insert, at 2 hours post-seeding, suggesting both were properly cross-linked (Figure 3A). After 48 h in culture, GL/SA embedded cells display similar pattern whereas in fibrin gels, cells were all confined to the insert membrane surface (Figure 3B). These data demonstrated that MRC-5 cells degrade only fibrin hydrogels within two days leading to cell sedimentation. We assumed this was also the case in GAF although we did not prove it directly in this work.

### MRC-5 cells secrete fibrinolytic serine proteases to degrade gelatin/alginate/fibrin hydrogels

**FIGURE 4:**
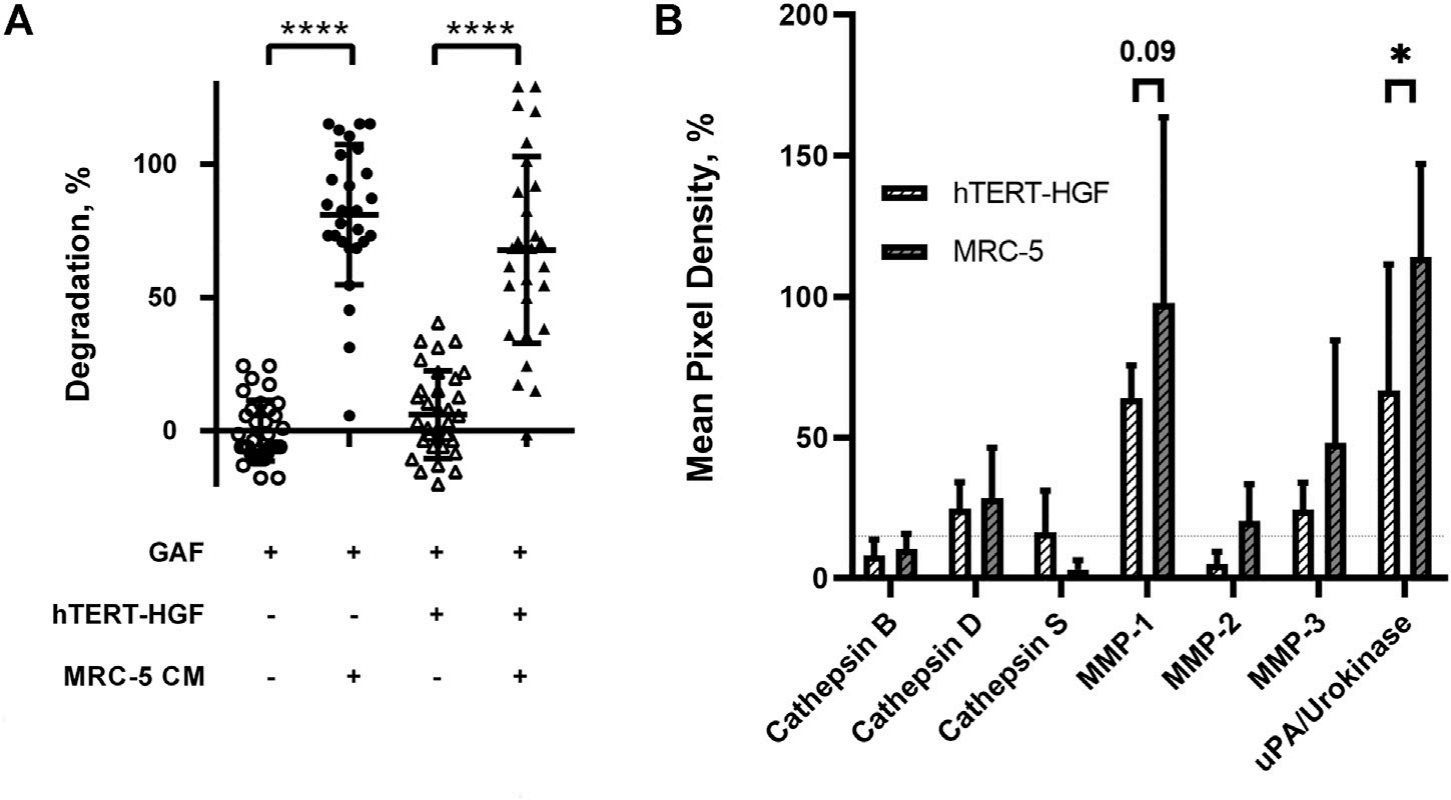
Fibrinolytic proteases are released in MRC-5 and hTERT-HGF culture supernatants. **(A)** MRC-5 conditioned medium or MCR-5 CM promotes GAF degradation with (triangle) or w/o hTERT-HGF (circle). Degradation was assessed at 72 h by image analysis of light transmission as described in Figure 1 and Materials and Methods. **(B)** Secreted serine proteases analyses in culture medium of 3D-cultured hTERT-HGF (dashed white) and MRC-5 cells (dashed gray). Values are represented as percentages of Mean Pixel Density ± SD in positive controls. Threshold was set at 15% (horizontal thin dashed line). Statistically significant results were marked by one or several asterisks according to the level of significance: *p < 0.05, ***p < 0.001 and ****p < 0.0001; two-way ANOVA tests.

We then investigated whether the degradation was caused by soluble factors or cell interaction with the surrounding environment. Forty-eight-hour 3D MRC-5 culture supernatant, namely MRC-5 conditioned medium (CM) was collected and added to cell-free or hTERT-HGF-containing GAF scaffolds, both harboring no significant difference. After a 72 h incubation with CM, GAF and hTERT-HGF-containing gels underwent degradation (80.96 ± 26.27% and 67.79 ± 34.96%) demonstrating that CM contained fibrinolytic secreted factors (Figure 4A). No difference were seen between with or w/o hTER-HGF conditions suggesting that CM was the only cause for degradation. The individual value distributions reflect the heterogeneity in the ability to degrade GAF. To characterize the soluble factors involved, 2D and 3D culture supernatants from MRC-5 and hTERT-HGF were analyzed using specific antibody coated nitrocellulose membranes allowing for the detection of various secreted proteases. Mean Pixel Intensities (MPDs) were obtained from the analysis of digitalized images of several membranes. Data were normalized on negative and positive controls to infer the amount of secreted proteases through MPD values displayed in Figure 4B (and Supplementary Table S2). MPDs for all considered proteases in 3D-culture of MRC-5 cells in-gel tended to be or were superior to those of hTERT-HGF 3D supernatants, except for cathepsin S (3.1 ± 3.3% and 16.3 ± 14.7%, respectively), the most expressed being MMP-1, MMP-3 and urokinase for hTERT-HGF and MRC-5 cells (63.9 ± 11.6 vs*.* 97.7 ± 65.8, 24.4 ± 9.6 vs*.* 48.1 ± 36.4 and 66.7 ± 44.8 vs*.* 114 ± 33.1, respectively), all three of which are known to be directly or indirectly involved in fibrinolysis (Kluft, 2003; Lijnen, 2006). Urokinase expression was statistically higher in MRC-5 3D supernatant then hTERT-HGF 3D supernatant and MMP-1 tended to be higher (*p*-value 0.09). To compensate the non-significant observed differences, we investigated the dynamic changes in protease secretion that may occur when converting 2D cultures into a 3D environment. To that purpose we compared protease secretion in 2D vs*.* 3D culture supernatants. Interestingly, hTERT-HGF displayed a decreased ability to secrete all considered proteases with significant decrease in levels for MMP-1 (122.3 ± 59.2% to 63.9 ± 11.6%) and MMP-2 (70.5 ± 13.4% to 5.1 ± 4.4%) except for urokinase (2.9 ± 2.6% to 66.7 ± 44.8%) when moved to in-gel cultures (Supplementary Figure S2). Similarly, MRC-5 cells tended to secrete more of them in 3D conditions (Supplementary Figure S2) with significant decrease of gelatinase MMP-2 levels (73.2 ± 27.8% and 20.4 ± 13.0%) but no consequence on GAF degradation documented in that setting. Altogether, these results suggested that 2D cultures secreted large amounts of proteases with fibrinolytic activities like urokinase and possibly MMP-1- while 3D-cultured MCR-5 cells promoted in turn the fibrin degradation of GAF hydrogels.

### Simultaneous targeting of plasmin and matrix-metalloproteinases activities prevent fibrin degradation

**FIGURE 5:**
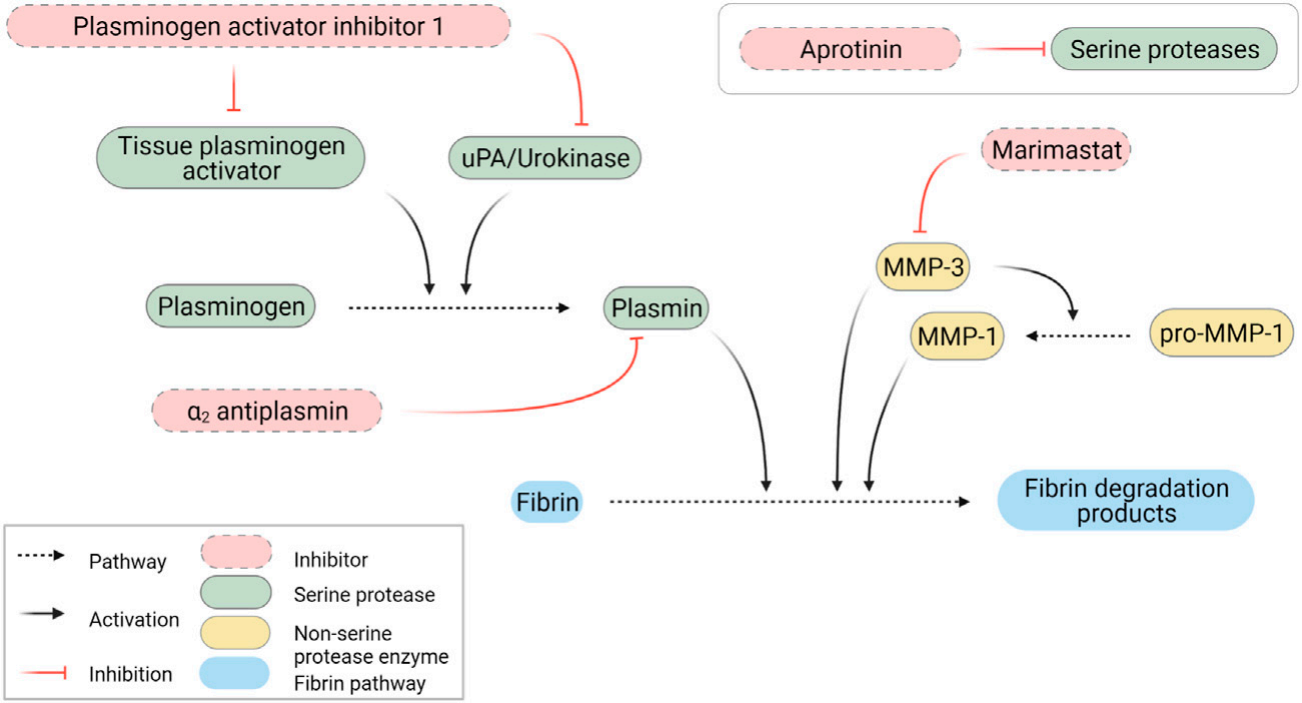
Schematic representation of fibrinolytic pathways. Created with BioRender.com.

**FIGURE 6:**
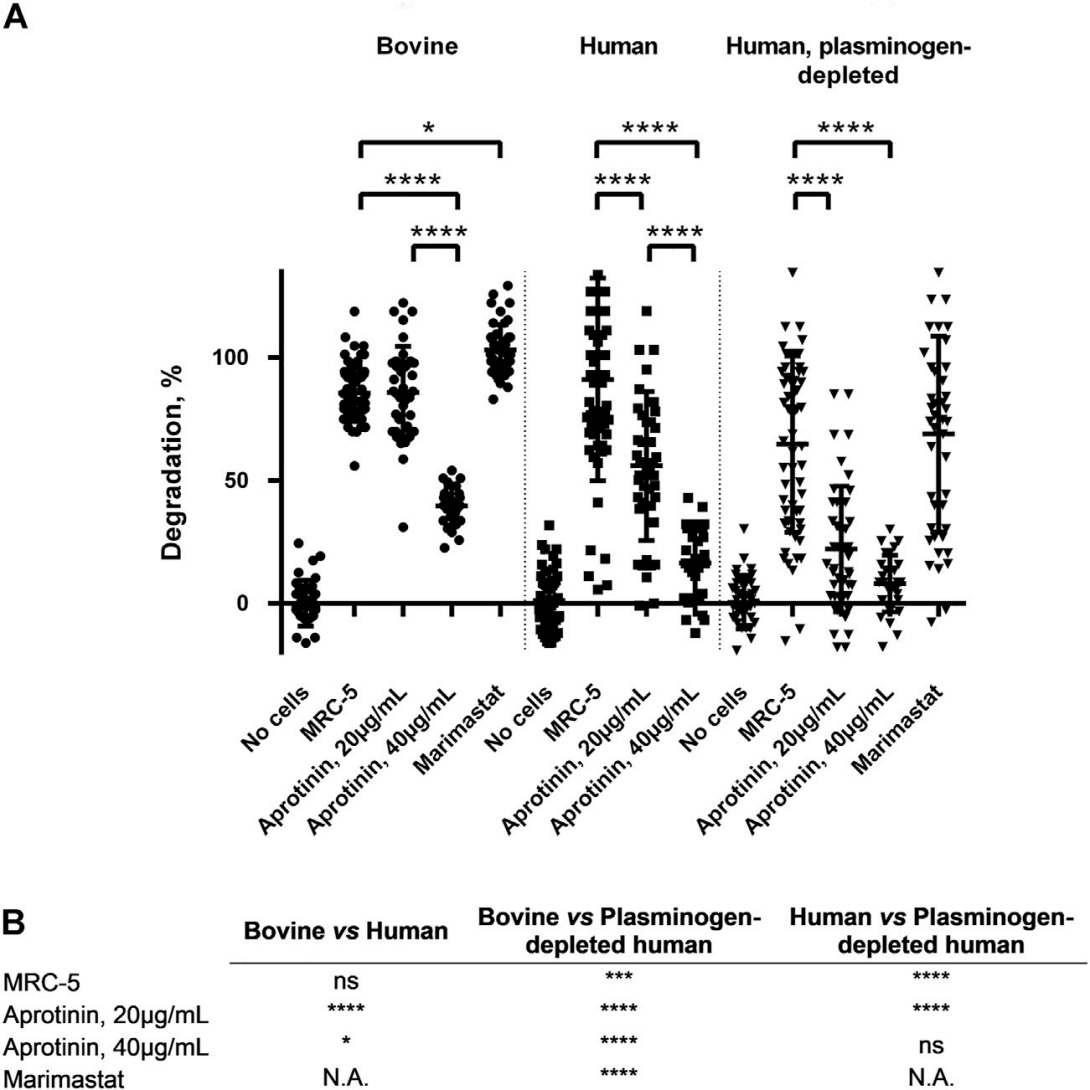
GAF degradation inhibition by MMPs and/or plasmin inhibitors or using plasminogen-depleted FBG. **(A)** MRC-5 cell-containing GAF prepared with either bovine (circle), human (square) or human plasminogen-depleted FBG (triangle) were treated with aprotinin (20 µg/mL or 40 µg/mL) or Marimastat (100 µM). GAF degradation was assessed as described in Materials and Methods section. Individual values are plotted, *n* ≥ 30. Statistically significant results were marked by one or several asterisks according to the level of significance: *p < 0.05, **p < 0.01, ***p < 0.001 and ****p < 0.0001; Tukey’s multiple comparisons tests. **(B)** Statistical significance of the comparisons between various experimental conditions shown in **Panel A**.

Based on the secreted protease profiling of MRC-5-containing degraded scaffolds we next evaluated whether we could slow down GAF degradation. Urokinase acts on plasmin activation by cleaving its precursor, plasminogen, and a well-known contaminant of FBG extracted from bovine and human plasmas (Markus and Ambrus, 1960; Yaron et al., 2021) (Figure 5). We compared the stability of MRC-5 cell-containing GAFs prepared with bovine, human and plasminogen-depleted human FBGs. None of them remained stable after a 48-h incubation (85.5% ± 11.4%, 94.7 ± 42.11% and 64.8% ± 35.9% degradation, respectively). Noticeably, both human FBGs displayed heterogeneous degradation compared to bovine FBG with no clear explanation, all three exhibiting similar activities ranging between 90% and 95% clottable protein contents (Figure 6). Moreover, plasminogen depletion significantly decreased degradation (up to 29.8% ± 4.5% reduction). Then, we tested the aprotinin, a broad serine protease inhibitor, to counteract the degradation process. At 20 μg/ml, inhibition of degradation was observed only when used with human FBG (38.8% ± 5.0% reduction) and more potent with plasminogen-depleted human FBG (42.8 ± 5.0% reduction). As expected, at a higher dose (40 μg/ml), aprotinin inhibited GAF degradation (39.5% ± 7.6% for bovine FBG, 16% ± 14.8% for human FBG and 8.0% ± 11.5% plasminogen-depleted human FBG), demonstrating a dose-effect of aprotinin on fibrin degradation. To further increase evidence that plasmin and fibrinolytic MMPs were involved in GAF degradation, we next assessed the addition of Marimastat to plasminogen-depleted FBG-based GAF for 48 h. Results showed that the use of Marimastat did not significantly improve the stability of hydrogel using plasminogen-depleted human FBG (Figure 6). These data demonstrated significant and consistent inhibition of GAF degradation by aprotinin. Moreover, the use of plasminogen-depleted human FBG has shown some reduction in GAF degradation compared to human FBG suggesting it could be used without aprotinin to control the degradation of gels embedding cells with low or no fibrinolytic activities.

## Discussion

In tissue engineering, choosing appropriate cell types is of paramount importance as suggested by Langer and Vacanti several decades ago (Langer and Vacanti, 1993). This was also highlighted more recently by Manita et al. (2021) by focusing on the impact of cells on engineered skin stability and composition changes over time. Achieving assembly of tissues containing multiple cell types could be even more challenging due to intercellular cross-talk or interference between 3D matrix components and cells. In this study, we identified cellular determinants of fibrin network degradation in a blended biomaterial made of gelatin, sodium alginate and fibrin or GAF by transcriptionally distinct human fibroblastic cell types exhibiting a functional heterogeneity towards fibrin degradation. Previous work has described the use of GAF to support normal skin fibroblast (Reijnders et al., 2015; Pourchet et al., 2017) 3D cultures without degradation, highlighting the necessity to select non-degradative cells as a first option.

Fibrin degradation has long been described as a critical player in engineered tissue remodeling (Neuss et al., 2010). On the other hand, fibrinolysis impairs 3D matrix stability, thus limiting the use of fibrin with cells that actively remodel or secrete factors that promote degradation, like primary gingival or dermal fibroblasts Lorimier et al. (1996). Here, we demonstrated that degradation was due to an enhanced secretion of fibrinolytic MMPs and urokinase by GAF-embedded MRC-5 cells in line with previous studies. Interestingly, Ahmed et al. (2007) reported a direct upregulation of MMPs when 2D cell cultures were shifted to 3D. Here, we showed that the MRC-5 degradative potential towards GAF was mediated by soluble factors secreted in the culture medium and active on cell-free as well as on hTERT-HGF-laden GAF within the first 24 h. These results confirmed a previous observation showing that MRC-5 CM can increase invasion and migration capacities of the MHCC-LM3 cell line, a hepatocellular carcinoma, through a non-classical epithelial-to-mesenchymal transition pathway (Ding et al., 2015). Here, we showed a possible link between the fibrinolytic activity of various fibroblastic cell lines and the *SHOX-2* gene expression levels, i.e., low expression being observed in highly degradative cells, namely MRC-5 and primary HGF. Interestingly, Hsia and colleagues demonstrated that *SHOX-2* expression is high to moderate in skin fibroblasts or activated fibroblasts respectively, but almost absent from myofibroblasts in cancer and normal tissues (Hsia et al., 2016). Although a high *SHOX-2* expression was correlated to enhanced migration and invasion capacities for breast cancer cells through a SHOX-2/STAT3/WASF3 axis, no relation has been established between the *SHOX-2* expression and fibrinolysis (Teng et al., 2021). In this work, we did not compare the *SHOX-2* expression of the tested cell lines to myofibroblasts. However, we noticed a lower *SHOX-2* expression in fibrinolytic cells. Further studies are required to establish whether *SHOX-2* plays a role in fibrinolysis.

In this study, MRC-5 cells and primary HGF were shown to degrade GAF through fibrinolysis induction. Fibrinolysis is mediated through serine proteases, mainly MMP-3 and plasmin generated from urokinase- or tissue plasminogen activator (tPA)-activated plasminogen. Several studies have investigated the possibility to extend the stability of adipose-stromal cell (Xu et al., 2009) or HeLa cell-laden (Zhao et al., 2014) fibrin-containing matrices using aprotinin, a non-specific protease inhibitor targeting plasmin, tPA, urokinase and MMPs among other proteases. Aprotinin was previously used alone or in combination with galardin, a wide spectrum MMPs inhibitor (Ahmed et al., 2007). Ye et al. demonstrated that cardiac myofibroblasts would degrade fibrin matrices in two days (Ye et al., 2000) while no degradation was observed for a month using aprotinin at 15–20 μg/ml (Lesman et al., 2011). Similar results were also reported by Thompson et al. (2013) on a cardiac tissue model. Studies focusing on Duchenne muscular dystrophy (Gerard et al., 2012), metabolic syndrome (Xu et al., 2010) or 3D-bioprinted tissues with matrix-remodeling cells (Kang et al., 2016) reported addition of aprotinin to preserve hydrogel or bioink integrity in the course of long-lasting experiments without investigating potential phenotypic or functional changes for cells. Aprotinin also showed efficacy in blocking the degradation of electrospun fibrin fibers (McManus et al., 2006) as well as cell-laden blended hydrogels to obtain various tissues like cartilage (Lee et al., 2005), cornea (Foroushani et al., 2021), retina (Gandhi et al., 2018) and CNS stroma (Seyedhassantehrani et al., 2016). Finally, molar excess of aprotinin has proven efficacy in slowing down fibrinolysis of commercial homologous fibrin surgical sealants made of human plasma-purified FBG and activated factor XIII cross-linked with thrombin and calcium ions (Buchta et al., 2005). Ahmann and colleagues previously proposed defined concentrations of ɛ-aminocaproic acid (ACA) to achieve similar results (Ahmann et al., 2010). ACA inhibits urokinase (Bakker et al., 1995) and plasmin (Anonick et al., 1992) and has been widely used also in tissue engineering to slow down fibrin degradation (Ramaswamy et al., 2019). With regards to the broad usage of aprotinin or ACA to stabilize engineered fibrin-based matrices, very few adverse effects were reported to date when inhibitor concentrations are optimized. One example was brought by Kim et al. (2015) by showing that aprotinin concentrations above 0.05 TIU/ml allowed HS-5 cells, a human bone marrow stromal cell line, to grow and remodel their fibrin-based matrix further leading to T-cells invasion and establishment of heterotypic cell-to-cell interactions. One exception concerns an observation made by Mühleder et al. (2018) who demonstrated that aprotinin supplementation could impair vessel formation without affecting branching or ECM deposition in an engineered vascular network model. Although few alterations of cellular functions have been documented to date when using aprotinin, we believe that comprehensive studies on the impact of protease inhibitor treatments on various cell types would be necessary to fully estimate their optimal concentrations to limit fibrinolysis without impairing cellular functions.

In addition to aprotinin supplementation to strongly delay GAF degradation, we demonstrated that hTERT-HGF or FSF did not display intrinsic fibrinolytic activity making them suitable for fibroblast embedding in fibrin-based matrices. In association with immortalized gingival keratinocytes, hTERT-HGF were shown to reconstitute a stable full-thickness gingiva (Buskermolen et al., 2016). A similar approach with immortalized cells from skin origin led to the generation of a full-thickness skin equivalent (Reijnders et al., 2015). However, fibroblasts were embedded in collagen-based hydrogels devoid of fibrin in both studies. In this study, we proved that using plasminogen-depleted FBG of human origin allowed for the fabrication of more stable MRC-5-containing hydrogels for at least two days, supporting the idea that plasmin and/or Marimastat-insensitive MMP inhibition leads to slow down fibrinolysis. We have tested broad inhibitor for MMP and serine proteases, however, we did not evaluate the activity of proteases in the supernatant, nor the effect of addition to FCS to the medium in terms on addition of proteases or their possible excess compared to inhibitors quantities. Similar results were obtained by Jarrell et al. (2021) who demonstrated that increasing sodium chloride concentrations up to 250 mM, i.e., hyperosmotic, instead of 150mM, i.e., a physiological concentration, led to transparent fibrin gel formation degrading two to three times slower than controls. However, two limitations were foreseen in the study by Jarrell et al. (2021) when considering to apply this protocol to cell-laden hydrogels. First, hyperosmolar culture media or buffers can cause an osmotic shock leading to a rapid cell shrinkage and associated cell death. Then, surviving cells could undergo substantial transcriptomic changes, as shown for gingival fibroblasts, most notably altering genes whose products are involved in extracellular matrix remodeling potentially leading to undesirable effects (Schroder et al., 2019).

In summary, this study provides new insights to understand mechanisms behind GAF degradation by some human fibroblast sub-types. We confirmed that blocking proteases and plasmin is an efficient way to prevent fibrinolysis which drives GAF degradation. We also proposed to use a combination of plasminogen-depleted FBG and aprotinin to enhance the degradation slow down when utilizing fibrinolytic cells like MRC-5. In conclusion, we believe that our results will contribute to extend possibilities to engineer fibroblast-embedded CTs by emphasizing primarily on the choice for cells with no or low fibrin remodeling properties, and either as primary cultures or immortalized cell lines. HTERT-immortalized cell lines usually present the advantage of supporting long-lasting cultures with rather stable phenotypes. Therefore, they can support genetic modifications, e.g., genome editing, to establish experimental models for basic science and precision medicine.

## Data Availability

The raw data supporting the conclusions of this manuscript will be made available by the authors, without undue reservation.
