# Supplementary material for: The degradation of gelatin/alginate/fibrin hydrogels is cell type dependent and can be modulated by targeting fibrinolysis
Source: Front Bioeng Biotechnol. 2022 Jul 22;10:920929. doi: 10.3389/fbioe.2022.920929 (PMC9355319; doi:10.3389/fbioe.2022.920929)
Supplement: Supplementary file 1 [file Table1.docx]

**Supplementary Table 1:** List of genes analyzed by RT-qPCR in this study and related Taqman probes.

| Gene | Accession number | Probe name | Amplicon size |
| --- | --- | --- | --- |
| *AOC-3* | NM_001277731.1 NM_003734.3 NM_001277732.1 | Hs02560271_s1 | 94 pb |
| *ACTA2* | NM_001141945.2 | Hs00426835_g1 | 105 pb |
| *COLIA1* | NM_000088.3 | Hs00164004_m1 | 66 pb |
| *EGFR* | NM_005228.3 | Hs01076090_m1 | 57 pb |
| *FAP* | NM_001291807.1  NM_004460.3 | Hs00990791_m1 | 64 pb |
| *GAPDH* | NM_001289746.1 | Hs99999905_m1 | 122 pb |
| *PDGFRA* | NM_006206.4 | Hs00998018_m1 | 84 pb |
| *S100A4* | NM_002961.2 NM_019554.2 | Hs00243202_m1 | 101 pb |
| *SHOX-2* | NM_003030.4 NM_006884 NM_001163678.1 | Hs00243203_m1 | 129 pb |
| *THY-1* | NM_001311160.1 NM_001311162.1 NM_006288.4 | Hs06633377_s1 | 66 pb |
| *VIM* | NM_003380.3 | Hs00958111_m1 | 65 pb |
