# Supplementary figures and images for: The degradation of gelatin/alginate/fibrin hydrogels is cell type dependent and can be modulated by targeting fibrinolysis
Source: Front Bioeng Biotechnol. 2022 Jul 22;10:920929. doi: 10.3389/fbioe.2022.920929 (PMC9355319; doi:10.3389/fbioe.2022.920929)

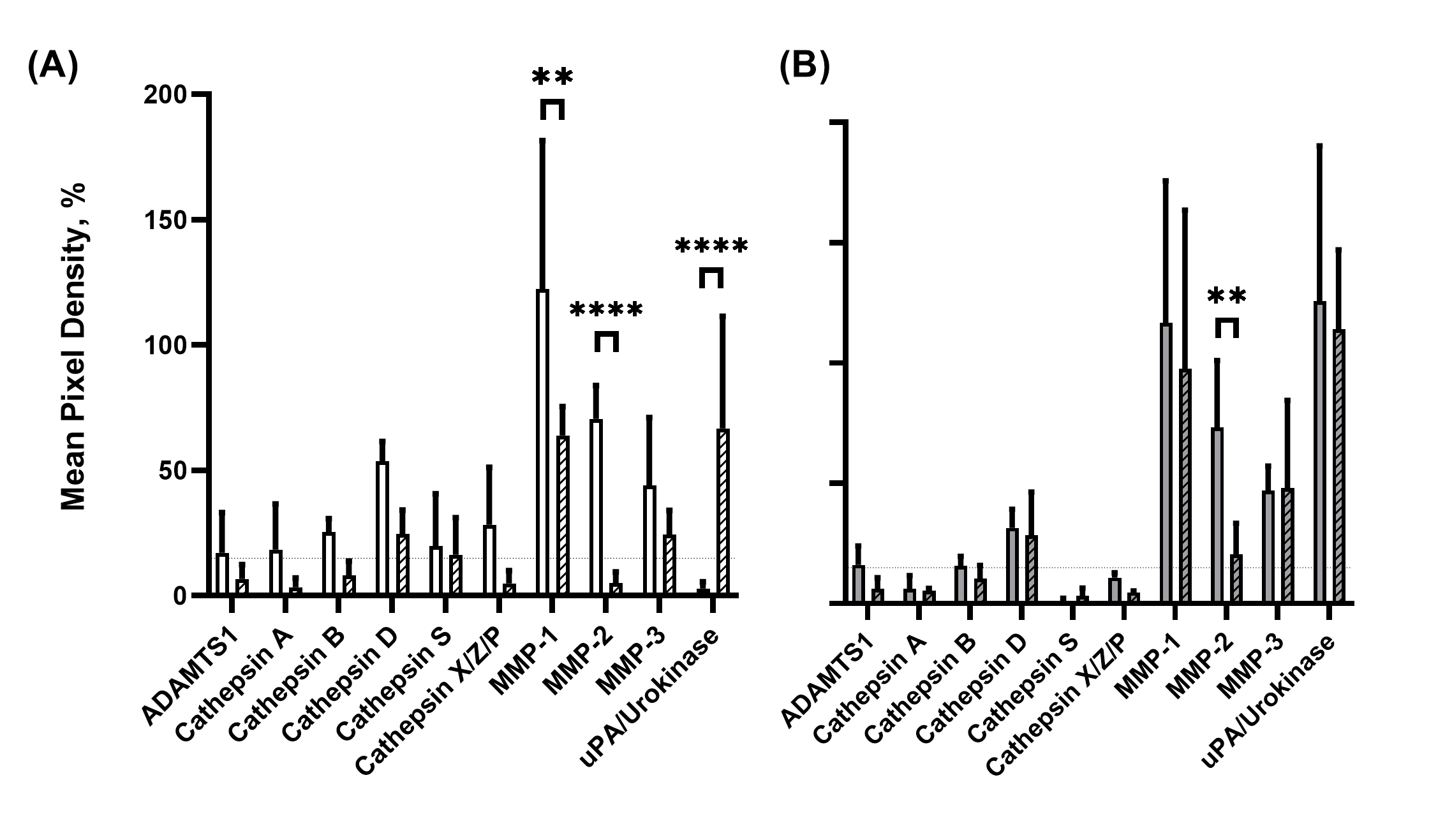

Supplement: Supplementary file 2 [file Image2.TIF]

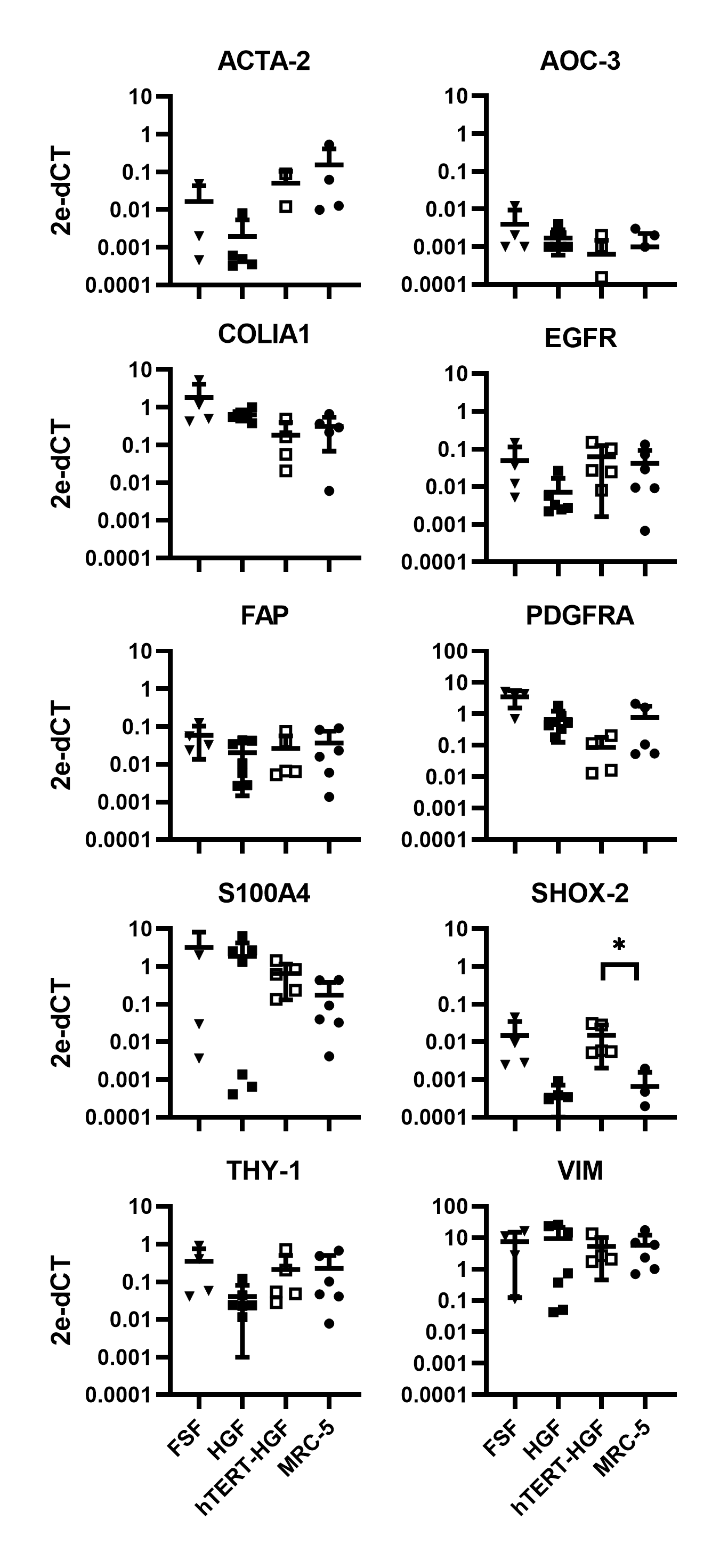

Supplement: Supplementary file 3 [file Image1.TIF]
