# Supplementary material for: The degradation of gelatin/alginate/fibrin hydrogels is cell type dependent and can be modulated by targeting fibrinolysis
Source: Front Bioeng Biotechnol. 2022 Jul 22;10:920929. doi: 10.3389/fbioe.2022.920929 (PMC9355319; doi:10.3389/fbioe.2022.920929)
Supplement: Supplementary file 4 [file Table2.docx]

**Supplementary Table 2:** Secreted serine protease profiles of hTERT-HGF and MRC-5, in 2D vs 3D culture conditions. Values are represented as percentages of mean pixel densities ±SD.

|  | | hTERT-HGF | | | MRC-5 | | |
| --- | --- | --- | --- | --- | --- | --- | --- |
|  | 2D | | 3D | 2D | | 3D |  |
| ADAM8 | 2.66 ± 2.87 | | 2.29 ± 1.96 | 0.71 ± 0.76 | | 0.95 ± 1.36 |  |
| ADAM9 | 12.5 ± 11.15 | | 6.95 ± 5.18 | 3.69 ± 3.43 | | 2.91 ± 3.19 |  |
| ADAMTS1 | 17.15 ± 16.01 | | 6.67 ± 5.74 | 16.07 ± 7.95 | | 6.07 ± 4.63 |  |
| ADAMTS13 | 1.32 ± 1.19 | | 0.74 ± 0.77 | 0.45 ± 0.47 | | 0.27 ± 0.19 |  |
| Cathepsin A | 18.37 ± 18.19 | | 3.35 ± 3.64 | 6.2 ± 5.42 | | 5.33 ± 0.98 |  |
| Cathepsin B | 25.35 ± 5.33 | | 8.03 ± 5.77 | 15.73 ± 3.95 | | 10.37 ± 5.48 |  |
| Cathepsin C | 4.87 ± 4.52 | | 0.88 ± 0.73 | 3.26 ± 2.65 | | 1.16 ± 1.24 |  |
| Cathepsin D | 53.53 ± 7.94 | | 24.61 ± 9.53 | 31.51 ± 7.75 | | 28.36 ± 17.92 |  |
| Cathepsin E | 1.54 ± 1.52 | | 2.11 ± 1.67 | 0.68 ± 0.65 | | 0.91 ± 0.87 |  |
| Cathepsin L | 6.21 ± 6.52 | | 4.25 ± 3.17 | 2.12 ± 1.97 | | 1.84 ± 2.05 |  |
| Cathepsin S | 19.79 ± 20.87 | | 16.31 ± 14.72 | 1.21 ± 1.06 | | 3.09 ± 3.34 |  |
| Cathepsin V | 10.51 ± 5.73 | | 8.73 ± 6.95 | 4.72 ± 1.78 | | 5.02 ± 2.17 |  |
| Cathepsin X/Z/P | 28.35 ± 22.8 | | 4.83 ± 5.11 | 10.78 ± 2.06 | | 4.6 ± 0.62 |  |
| DPPIV/CD26 | 6.03 ± 6.57 | | 1.56 ± 1.28 | 5 ± 2.99 | | 4.5 ± 1.45 |  |
| KLK3 | 1.87 ± 1.56 | | 0.67 ± 0.33 | 1.19 ± 1.05 | | 0.42 ± 0.52 |  |
| KLK5 | 2.85 ± 2.28 | | 1.52 ± 1.23 | 1.62 ± 1.04 | | 0.8 ± 1.08 |  |
| KLK6 | 1 ± 0.84 | | 1.58 ± 1.27 | 0.53 ± 0.48 | | 0.62 ± 0.89 |  |
| KLK7 | 1.68 ± 1.44 | | 2.33 ± 1.86 | 0.72 ± 0.55 | | 1.07 ± 0.94 |  |
| KLK10 | 3.21 ± 3.08 | | 2.95 ± 2.85 | 0.65 ± 0.59 | | 0.8 ± 0.94 |  |
| KLK11 | 1.32 ± 0.98 | | 1.66 ± 1.43 | 0.25 ± 0.6 | | 0.39 ± 0.34 |  |
| KLK13 | 4.14 ± 3.86 | | 1.66 ± 1.57 | 1.3 ± 1.29 | | 0.9 ± 1.02 |  |
| MMP-1 | 122.33 ± 59.2 | | 63.91 ± 11.58 | 116.76 ± 58.99 | | 97.72 ± 65.79 |  |
| MMP-2 | 70.5 ± 13.35 | | 5.06 ± 4.37 | 73.23 ± 27.79 | | 20.38 ± 12.94 |  |
| MMP-3 | 43.88 ± 27.12 | | 24.4 ± 9.6 | 46.96 ± 10.24 | | 48.09 ± 36.42 |  |
| MMP-7 | 3.87 ± 2.96 | | 3.76 ± 2.89 | 2.02 ± 1.19 | | 2.92 ± 3.59 |  |
| MMP-8 | 3.73 ± 3.8 | | 5.06 ± 3.96 | 0.82 ± 0.53 | | 2.37 ± 2.95 |  |
| MMP-9 | 2.18 ± 2.1 | | 2.95 ± 2.85 | 0.65 ± 0.73 | | 0.88 ± 1.46 |  |
| MMP-10 | 1.42 ± 1.17 | | 0.99 ± 1.02 | 0.64 ± 0.67 | | 2.69 ± 3.21 |  |
| MMP-12 | 2.89 ± 2.27 | | 0.81 ± 0.84 | 0.94 ± 0.82 | | 0.53 ± 0.37 |  |
| MMP-13 | 4.32 ± 3.87 | | 2.59 ± 1.78 | 2.21 ± 2.15 | | 1.15 ± 1.14 |  |
| NEP/CD10 | 8.26 ± 7.8 | | 1.58 ± 1.33 | 1.2 ± 1.15 | | 0.64 ± 0.49 |  |
| PSEN1 | 2.1 ± 1.95 | | 0.42 ± 0.35 | 1.04 ± 0.93 | | 0.19 ± 0.4 |  |
| PCSK9 | 0.57 ± 0.45 | | 0.68 ± 0.38 | 0.47 ± 0.56 | | 0.47 ± 0.21 |  |
| PRTN9 | 0.65 ± 0.56 | | 2.02 ± 1.57 | 0.41 ± 0.82 | | 0.97 ± 0.9 |  |
| uPA/Urokinase | 2.91 ± 2.58 | | 66.7 ± 44.78 | 125.77 ± 64.45 | | 113.99 ± 33.1 |  |
