# Supplementary material for: The degradation of gelatin/alginate/fibrin hydrogels is cell type dependent and can be modulated by targeting fibrinolysis
Source: Front Bioeng Biotechnol. 2022 Jul 22;10:920929. doi: 10.3389/fbioe.2022.920929 (PMC9355319; doi:10.3389/fbioe.2022.920929)
Supplement: Supplementary file 5 [file DataSheet1.docx]

Supplementary Material

# Supplementary Figures and Tables

**Supplementary Figure 1.** Gene expression analysis in FSF, HGF, hTERT-HGF and MRC-5 cells for *ACTA-2*, *AOC-3*, *FAP*, *EGFR*, *COLIA1*, *THY-1*, *PDGFRA*, *SHOX-2* and *S100A4* in standard 2D in vitro cultures by RT-qPCR. Performed similarly to experiments displayed in Figure 2 except that values were normalized to GAPDH. Statistically significant results were marked by one or several asterisks according to the level of significance: *p<0.05; Kruskal-Wallis tests.

**Supplementary Figure 2.** Secreted serine proteases analyses in culture medium of 3D-cultured hTERT-HGF (empty dashed; *vs* 2D cultures as empty bars) and MRC-5 cells (dashed gray; *vs* gray bars). Values are represented as percentages of Mean Pixel Density ± SD in positive controls. Threshold was set at 15% (horizontal thin dashed line). Statistically significant results were marked by one or several asterisks according to the level of significance: *p<0.05, ***p<0.001 and ****p<0.0001; two-way ANOVA tests.

**Supplementary Table 1.** List of genes analyzed by RT-qPCR in this study and related Taqman probes and amplicon size for each probe.

**Supplementary Table 2.** Secreted serine protease profiles of hTERT-HGF and MRC-5, in 2D *vs* 3D culture conditions. Values are represented as percentages of Mean Pixel Densities ±SD.
